# 596 Implementation of a Burn Laser Program at a Children’s Hospital

**DOI:** 10.1093/jbcr/irac012.224

**Published:** 2022-03-23

**Authors:** Nicole M Kopari, Kristen Lindsey, Herb A Phelan, Jeffrey E Carter

**Affiliations:** Children's Hospital New Orleans, New Orleans, Louisiana; Children's Hospital New Orleans, New Orleans, Louisiana; LSUHSC-New Orleans Department of Surgery, New Orleans, Louisiana; University Medical Center- New Orleans, New Orleans, Louisiana

## Abstract

**Introduction:**

Carbon dioxide ablative fractional laser (CO2-AFL) therapy has become standard of care for adult burn hypertrophic scars (HTS). This therapy option has not been widely adopted in pediatric burn care and no established guidelines for treatment protocols have been published. We sought to modify our American Burn Associated Adult Verified Burn Centers laser protocol at our Children’s Hospital with hopes to provide optimal care to our pediatric burn population. We present our protocol and early experience of CO2-AFL therapy for pediatric burn HTS.

**Methods:**

We conducted a retrospective chart review of pediatric burn patients undergoing CO2-AFL treatment of HTS during the study period of Jan 2021-Oct 2021. Pediatric burn patients were offered laser treatment if their scars were symptomatic with patient complaints of HTS, pruritis, neuropathic pain, and scar contractures. 37 pediatric patients ≤13 years of age were included in our review.

**Results:**

We treated 13 pediatric patients for a total of 40 laser sessions with each patient averaging 3 sessions. Of the 13 patients that were treated with laser, 62% (8 of the 13 patients) had split-thickness skin grafting with 38% (3 of the 8 patients) of those having a staged grafting procedure with dermal substitute. 15% (2 of the 13 patients) healed primarily and 15% (2 of the 13 patients) required excision and closure. Only 1 patient treated with ASCS alone required laser therapy. Our protocol requires patients to receive pre-operative Tylenol, Benadryl, Pepcid, and Oxycodone. The patients then received MAC anesthesia with Toradol, Dexamethasone, Ketamine or Propofol, and Zofran. Patients with extensive HTS on the face or neck were intubated for the procedures. Oxycodone and/or Dilaudid were provided if needed in the post-operative phase. All patients were discharged with Tylenol or Motrin and Triamcinolone 0.1% ointment to be applied daily for 48 hours and then 3-4x/day until the follow-up clinic appointment at one week. Patients were able to resume normal activities the day following the procedure.

**Conclusions:**

Patients and their parents have reported improvements in pigment, pliability, thickness, and pruritis following laser treatments. We created a protocol that allows on average 8 pediatric patients per day to receive laser treatment without it over burdening the pre-operative and post-operative recovery room nursing staff. We are currently tracking outliers of patients requiring increased post-operative analgesia and/or greater than 1 hour in the recovery phase. With the implementation of a laser protocol, we have successfully introduced laser therapy as a viable option for our pediatric burn survivors.

